# FSGS and COVID-19 in Non–African American Patients

**DOI:** 10.34067/KID.0000000000000104

**Published:** 2023-05-25

**Authors:** Elba Medina, Carlos Rueda, Daniel Batlle

**Affiliations:** 1 Division of Nephrology, General Hospital of México, Eduardo Liceaga, México City, México; 2 Master's and PhD Program in Dental and Health Medical Sciences, Universidad Nacional Autónoma de México, México City, México; 3 Division of Nephrology/Hypertension, Department of Medicine, Feinberg School of Medicine, Northwestern University, Chicago, Illinois

**Keywords:** FSGS, collapsing, *APOL1*, COVID-19, SARS-CoV-2, kidney biopsy

## Abstract

Collapsing Focal Segmental Glomerulosclerosis (FSGS) has been reported relatively frequently in African American (AA) patients with coronavirus disease 2019 (COVID-19), and it is associated almost always with Apolipoprotein L gen 1 (*APOL1*) high-risk variants. We reviewed the published literature from April 2020 to November 2022 searching for non–African American (non-AA) patients with FSGS associated with COVID-19 (eight White patients, six Hispanic patients, three Asian patients, one Indian patient, and one Asian Indian patient). The following histologic patterns were found: collapsing (*n*=11), not otherwise specified (*n*=5), tip (*n*=2), and perihilar (*n*=1). Fifteen of the 19 patients had AKI. The *APOL1* genotype was reported in only six of the 19 non-AA patients. Three of them (two Hispanic patients and one White patient) with collapsing FSGS had high-risk *APOL1* variants. The other three patients (two White patients and one Hispanic patient with the collapsing variant, tip variant, and not otherwise specified) had low-risk *APOL1* variants. Among 53 African American patients with collapsing FSGS associated with COVID-19, 48 had high-risk *APOL1* variants and five had low-risk *APOL1* variants. We conclude that in non-AA patients, FSGS is a rare complication of COVID-19. FSGS associated with COVID-19 can occur rarely with low-risk *APOL1* variants in non-AA and AA patients. Non-AA patients reported to be associated with high-risk *APOL1* variants possibly reflect inaccuracy of self-reported race with AA admixture because of unknown ancestry. Given the importance of *APOL1* in the pathogenesis of FSGS associated with viral infection and to avoid racial bias, it seems appropriate that *APOL1* testing be considered in patients with FSGS associated with COVID-19, regardless of self-reported race.

## Introduction

In patients with coronavirus disease 2019 (COVID-19) and clinical signs of kidney involvement, kidney biopsy usually shows features of acute proximal tubular injury consistent with the AKI clinical presentation.^1–4^ However, FSGS, particularly of the collapsing variant, has been reported relatively frequently in African American (AA) patients with COVID-19.^5–14^ Noncollapsing variants of FSGS have also been reported, albeit rarely.^1^ Collapsing glomerulopathy is often associated with certain viral infections, such as HIV.^15^ This glomerulopathy when linked to COVID-19 has been termed COVID-19 associated nephropathy (COVAN) by Velez *et al*.^16^ Histologically, there are no apparent differences between collapsing glomerulopathy caused by HIV and COVID-19 infections.^17^

Approximately 91%–100% of AA patients with COVID-19 with collapsing glomerulopathy carry high-risk Apolipoprotein L gen 1 (*APOL1*) variants.^1,9^ The *APOL1* risk genotypes, defined by the G1 and G2 risk alleles, are believed to be absent in populations without African ancestry and likely to have evolved to protect carriers in West Africa from African sleeping sickness (trypanosomiasis).^18,19^ The dispersion of people from West and Central Africa to the Americas and the Caribbean islands because of the trans-Atlantic slave trade and more recent migration has led to the global distribution of *APOL1* variants.^19^ Some studies identified two types of Hispanics: the continental one from mainland Latin America and the other from the Caribbean, with the latter group having an increased frequency up to 10 times higher of carrying *APOL1* risk alleles (0.1% vs 1%).^20^ Very little is known about the occurrence of FSGS in non-AA patients, and the impression is that this is a rare lesion because *APOL1* high-risk variants are usually absent.^7,21^ The purpose of this review was to survey the literature for non-AA patients with FSGS since the COVID-19 pandemic started and examine the characteristics of such patients and, when possible, their association with *APOL1* variants.

*APOL1* is a gene that represents an important risk factor for the development of podocytopathies, mainly in those with collapsing glomerulopathy and of African ancestry.^7,22^ Cell culture studies suggest that *APOL1* variants cause cell dysfunction through several processes, including alterations in cation channel activity—the G1 and G2 variants form cytotoxic cation channels at the surface of cells, which triggers an influx of Na+ and Ca+ across the plasma membrane and leads to cell death.^23,24^ Other mechanisms are inflammasome activation, increased endoplasmic reticulum stress, activation of protein kinase R, mitochondrial dysfunction, and disruption of *APOL1* ubiquitination.^18,24^

*APOL1* is encoded in the gene *APOL1* (locus 22q12.3). G0 is called the reference sequence *APOL1* allele or wild-type.^25^ In the kidney, G0 expression contributes to maintaining podocyte phenotype and integrity.^24^ There are two genetic variants for two different isoforms of the *APOL1*, G1 and G2, that are associated with kidney disease,^26^ FSGS,^27^ HIV nephropathy,^28^ and other forms of nondiabetic kidney disease in patients of African ancestry.^29^ We analyzed cases of FSGS reported in association with COVID-19 infection from the beginning of the pandemic until November 2022. We were able to review individual data from 19 non–African American patients. In addition, data were included from 94 African American patients (for the purpose of this review article by an African American, we included Black patients from the United States, Africa, and worldwide) with FSGS who had a kidney biopsy after COVID-19 infection. From the individual data available, we examined clinical characteristics and *APOL1* status when available in non-AA and AA patients.

## Methods

We performed a PubMed search from December 2019 to November 13, 2022 with the words “COVID-19 and biopsy and kidney,” “glomerulosclerosis and COVID-19,” “COVID-19 and FSGS,” “Collapsing glomerulopathy and COVID-19,” and “MCD and COVID-19” and found 1425 articles. Conference abstracts from the American Society of Nephrology (ASN) (2020–2022) and the European Renal Association (2020–2022) were also reviewed. From those, we identified a total of only 19 non–African American patients with FSGS of any variant. In this search, an additional seven patients with minimal change disease were identified who were not included in our analysis, except for one who converted to FSGS during COVID-19.

In all articles describing patients with information on *APOL1* status, we noted whether they had high-risk or low-risk genotype variants for FSGS. In total, this information was available in 55 of 94 African American patients (here referring to Black people from the United States, Africa, and worldwide). Patients reported as non–African American were specified as White, Hispanic, Asian, or Indian; when it was not specified, the patients were assumed to be non-AA because the reports came from China (*n*=2) and Croatia (*n*=3). Of the 19 non–African American patients, only six had *APOL1* status reported.

In transplant patients, we considered *APOL1* genotype from the donor as the risk factor, although it is still controversial which confers the most risk between the donor and recipient genotypes. We did not include patients from large series where ancestry was mentioned as a percentage of patients, but two studies^1,2^ are mentioned in the discussion as they had a significant number of non-AA patients.^1,2^

## Results

### Non–African American Patients

#### General Characteristics

In total, we found 19 non–African American patients with FSGS associated with COVID-19 reported in the literature (11 male and eight female): eight White patients, six Hispanic patients, three Asian patients, one Indian patient, and one Asian Indian patient. Each patient demographic and clinical characteristics are summarized in Table 1.

**Table 1:** FSGS and coronavirus disease 2019 infection in non–African American patients^a^

Patient	Sex	Age	Race	*APOL1*	FSGS (Type)	Glomerulopathy	History	AKI/Dialysis	COVID-19	Steroid	Transplant/Autopsy	Proteinuria	Serum Creatinine (mg/dl)	Time to Biopsy	Author
1	M	64 yo	Hispanic	N/A	Collapsing	*De novo*	DM, transplant source deceased	No/No	Severe	No	Yes/No	9.8 g/g	2.5	≤1 mo	Daniel^54^
2	M	42 yo	Indian	N/A	Collapsing	*De novo*	Previously healthy	No/No	Moderate	No	No/No	8 g/g	1.0	≤1 mo	Deshmukh^59^
3	M	48 yo	White	N/A	Collapsing	Progression	FSGS noncollapsing second renal transplant	Yes/No	N/A	N/A	Yes/No	11.51 g/g	2.65	≈1 mo	Thorburn^60^
4	F	56 yo	White	High-risk N/A	Collapsing	*De novo*	HTN, obesity, gastric bypass, hypothyroidism, and CKD	Yes/Yes	Mild	No	No/No	7.2 g/g	8.6	≤2 wk	Kudose^9^
5	M	69 yo	White		Collapsing	*De novo*	HTN, chronic lower extremity cellulitis, BPH, GERD, and CKD	Yes/Yes	Moderate	No	No/No	>1000 mg/dl	13.2	≤2 wk	
6	M	54 yo	Croatian	N/A	Collapsing	*De novo*	Nephroangiosclerosis, HTN, 2nd COVID-19 infection, and hepatitis B	Yes/No	Mild	No	Yes/No	7.44 g/d	N/A	N/A	Basic^61^
7	M	44 yo	Hispanic	G2/G2	Collapsing^b^	Relapsing	FSGS collapsing	Yes/Yes	Mild	No	No/No	11.4 g/g	12.00	6 wk	Akilesh^8^
8	M	41 yo	Hispanic	G0/G0	Collapsing	*De novo*	N/A	Yes/N/A	N/A	N/A	No/No	10.4 g/g	8.6	N/A	Nystrom^12^
9	F	51 yo	Hispanic	G1/G1	Collapsing	*De novo*	N/A	Yes/Yes	N/A	N/A	No/No	9.3 g/g	4.5	1.5 mo	
10	M	34 yo	White	Low-risk (NS)	NOS	*De novo*	HTN, anabolic steroids, and high-protein diet	Yes/No	Critical	Yes	No/No	26.5 g/d	6.45	17 d	Nowak^62^
11	F	56 yo	Hispanic	N/A	NOS	*De novo*	HTN, DLP, and depression	No/No	Mild	Yes	No/No	10.39 mg/g	0.63	N/A	Román^63^
12	F	87 yo	Chinese	N/A	NOS	*De novo*	DM, HTN, and CKD	Yes/No	Critical	No	No/Yes	3+ protein	N/A	N/A	Su^64^
13	M	87 yo	Chinese	N/A	NOS	*De novo*	DM, HTN, and CKD	Yes/No	Critical	No	No/Yes	N/A	2.6	N/A	
14	F	77 yo	Hispanic	N/A	NOS^b^	*De novo*	DM and HTN	Yes/Yes	Asymptomatic	No	No/No	13.41 g/g	3.99	3 d	Akilesh^8^
15	F	40 yo	Croatian	N/A	Tip	*De novo*	Lupus nephropathy and HTN^c^	Yes/Yes	Moderate	Yes	Yes/No	2.05 g/d	N/A	N/A	Basic^61^
16	F	20 yo	White	G0/G0	Tip	*De novo*	None	Yes/No	Mild	Yes	No/No	9 g/g	2.80	≤2 wk	Kudose^9^
17	M	60 yo	Croatian	N/A	Perihilar	*De novo*	Nephroangiosclerosis, HTN, and BKVAN**^c^**	No/No	Moderate	No	Yes/No	2.91 g/d	N/A	N/A	Basic^61^
18	M	71 yo	Asian Indian	N/A	1st B_x_: MCD 2nd B_x_: Collapsing	*De novo*	DM2, HTN, and BPH	Yes/Yes	Mild	Yes	No/No	18.46 g/g; 16g/d	4.49	1st B_x_: 21 d 2nd B_x_: 77 d	Gupta^65^
19	F	42 yo	Asian	N/A	Collapsing	*De novo*	None^d^	Yes/No	Asymptomatic	Yes	No/No	11 g	2.5	N/A	Ganglam^66^

*APOL1*, Apolipoprotein L gen 1; COVID-19, coronavirus disease 2019; M, male; N/A, not available; F, female; DM, diabetes mellitus; HTN, hypertension; BPH, benign prostatic hyperplasia; GERD, gastroesophageal reflux disease*;* NS, not specified; NOS, not otherwise specified; Protein; DLP, dyslipidemia; Urine Protein-Creatinine Ratio (g/g) or 24-hours urine protein (g/d) or dipstick proteinuria; B_x_, biopsy.; MCD, minimal change disease; BKVAN, BK virus–associated nephropathy (BK virus is an abbreviation of the name of the first patient, from whom the virus was isolated).

a The term “non–African American” included patients reported as non-Black from Asia, India, and Europe on the basis of the description used in the publications.

b TMA, thrombotic microangiopathy.

c Viral reactivation of Epstein-Barr virus detected in hospitalization.

d Crescentic glomerulonephritis and class IV lupus nephritis at the same time of diagnosis of the collapsing variant.

The mean age of patients was 55 (20–87) years. Ten of 19 patients had mild-to-moderate COVID-19, one severe, three critical, and two asymptomatic per the National Institutes of Health COVID-19 severity classification^30^; in the remaining three patients, the information was not available. Hypertension was the most prevalent comorbidity; one transplant patient had chronic hepatitis B infection and a history of an episode of COVID-19 3 months ago.

##### Renal Findings

Most of the kidney biopsies were performed within 4–6 weeks of a positive severe acute respiratory syndrome coronavirus 2 (SARS-CoV-2) PCR test. FSGS was reported in native kidneys (*n*=12), kidney allografts (*n*=5), and postmortem (*n*=2). *De novo* FSGS was observed in 17 patients, relapsing in one patient, and progression from minimal change disease to FSGS in the remaining patient.

Plasma creatinine was elevated (mean, 5.1±3.86 mg/dl; median, 3.99 mg/dl; range, 0.63–13.2). Fifteen of the 19 patients had AKI, and seven required hemodialysis. Nephrotic range proteinuria (defined as more than 3.5 g) was reported in 15 patients. Of the remaining four patients, three had 2.05, 2.91 g/d, and 3+ protein on a dipstick, in one patient (autopsy patient), the information on proteinuria was not available.

##### Type of FSGS and *APOL1* Status

**Figure 1:**
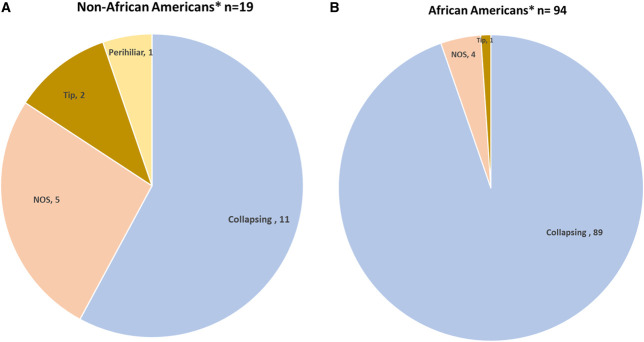
**FSGS variants and coronavirus disease 2019 in non–African American and African American patients.** (A) There were 19 non–African American patients: 11 with collapsing FSGS, five with NOS, one with the tip variant, and one with perihilar. (B) There were 94 African American patients: 89 were collapsing FSGS, four NOS, and one tip variant. *The term “non–African American” included patients reported from Asia, India, and Europe on the basis of the description in the articles, and “African American” included patients described as Black, including patients outside of the United States (see Methods). NOS, not otherwise specified.

**Figure 2:**
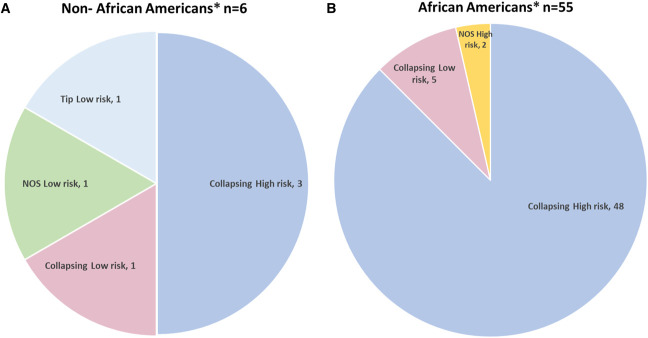
***APOL1* Genotype.** (A) In non-African Americans patients 6 were genotyped for *APOL1*; 3 had collapsing and APOL1-high risk, 1 had collapsing and APOL1-low risk; 1 had the tip variant and APOL1-low risk and the last patient had NOS and APOL1-low risk. (B) In African Americans patients 55 were genotyped for *APOL1*: 48 had collapsing and *APOL1*-high risk, 5 had collapsing and APOL1-low risk, and 2 had NOS and APOL1- high risk genotype. *The term “non–African American” included patients reported from Asia, India, and Europe, and “African American” included patients described as Black, including patients outside of the United States (see Methods). *APOL1*, Apolipoprotein L gen 1; NOS, not otherwise specified.

Eleven of the 19 non-AA patients had FSGS of the collapsing variant (Figure 1A). Eight had noncollapsing FSGS. *APOL1* was evaluated in six of the 19 non-AA patients (Figure 2A).

Of the patients with noncollapsing FSGS, five of eight had the not otherwise specified (NOS) variant as per the Columbia pathological classification of FSGS lesions.^31^ The tip variant was found in two patients and perihilar in one patient (Figure 1A, Table 1). In addition, one patient with the NOS variant had biopsy findings of thrombotic microangiopathy. *APOL1* was evaluated in only two of the eight patients with noncollapsing FSGS: one patient had a tip lesion and the other had NOS, both with *APOL1* low-risk variants.

One of the 11 AA patients with the collapsing variant had biopsy findings of thrombotic microangiopathy. *APOL1* was evaluated in four of the 11 non-AA patients with the collapsing FSGS variant: three patients had high-risk variants (one with G1/G1, one G2/G2, and it was not specified in one) and one patient with the collapsing variant had the low-risk variant G0/G0.

#### African American Patients

##### General Characteristics

We found a total of 94 AA patients (55 men, 34 women, five not specified), as summarized in Tables 2. The mean age of patients was 52±11 years (range 16–79 years). Of the 94 AA patients with COVID-19, 33 had mild disease, 25 moderate, 15 severe, nine critical, three asymptomatic, one was reported as hospitalized, and eight did not specify a score or any symptom per the National Institutes of Health COVID-19 severity classification.^30^

**Table 2:** FSGS associated with COVID-19 in African American patients^a^


Patient	Sex	Age	Race	*APOL1*	FSGS (Type)	Glomerulopathy	History	AKI/Dialysis	COVID-19	Steroid	Proteinuria	Serum Creatinine (mg/dl)	Time	Author
1	F	63	African	G1/G2	Collapsing	*De novo*	CKD 3, SLE, and APS	Yes/No	Mild	Yes	6.8 g/g	4.6	N/A	Hoilat^67^
2	M	64	African	G1/G0	Collapsing	*De novo*	HTN, DM, CKD 3, HIV controlled	Yes/Yes	Critical	Yes	2.74	2.3	11 d	Malhotra^37^
3	F	77	African	N/A	Collapsing	*De novo*	HTN, cardiac disease, and AFib	Yes/Yes	Severe	Yes	1.5 g/d	8.3	Third week	Sharma^68^
4	F	67	African	G1/G2	Collapsing	*De novo*	HTN, DM 2, DLP, OSA, and GERD	Yes/Yes	Mild	Yes	3276 mg/g	8.27	N/A	Sharma^14^
5	M	49	African	G1/G2	Collapsing	*De novo*	HTN, cardiomyopathy, peripheral vascular disease, and arthritis	Yes/Yes	Severe	Yes	2598 mg/g	10.1	N/A	
6	F	44	African	G1/G1	Collapsing	*De novo*	DM, HTN, DLP, and CKD	Yes/Yes	Severe	No	25 g/g	11.4	N/A	Larsen^69^
7	F	49	African	G1/G1	Collapsing	*De novo*	CKD, heart allograft, DM2, HTN, and obesity	Yes/No	Moderate	Yes	6.6 g/d	8.63	N/A	Izzedine^13^
8	F	38	African	G1/G1	Collapsing	*De novo*	CKD, HTN, obesity, and SLE (class IV glomerulonephritis)	No/No	Moderate	Yes	2.16 g/mmol	11.68	N/A	
9	F	63	African	G1/G1	Collapsing	*De novo*	HTN	Yes/No	Severe	No	5 g/L	8.4	8 d	Kissling^48^
10	M	46	African	Two with G1/G1 and one with G1/G2 the two patient's N/A	Collapsing	*De novo*	OSA and obesity	Yes/Yes	Mild	Yes	5.8 g/g o g/d	12.5	N/A	Kudose^5^
11	M	62	African		Collapsing	*De novo*	HTN, prostate carcinoma, and CKD	Yes/No	Mild	No	12.1 g/g o g/d	10.7	N/A	
12	M	62	African		Collapsing	*De novo*	HTN, DM, and prostate carcinoma	Yes/No	Moderate	Yes	19 g/g o g/d	11.6	N/A	
13	M	57	African		Collapsing	*De novo*	HTN and untreated hepatitis C virus	Yes/No	Moderate	No	6.2 g/g o g/d	4.9	N/A	
14	M	61	African		Collapsing	*De novo*	HTN and obesity	Yes/Yes	Mild	No	9 g/g o g/d	15	N/A	
15	M	64	African	G1/G2	Collapsing	*De novo*	Not known	Yes/No	Severe	No	7.6 g/d	7.2	≤1 mo	Shetty^10^
16	F	54	African	N/A	Collapsing	*De novo*	HTN	Yes/No	Mild	Yes	11.7 g/d	2.3	≤2 wk	
17	N/A	57	African	Rare *APOL1* genotype G1^GM^/G1^G+^	Collapsing	*De novo*	HTN	Yes/Yes	Mild	No	4.9 g/g	30.3	N/A	Laboux^70^
18	M	56	African	N/A	Collapsing	*De novo*	Ischemic cardiomyopathy and CKD and heart transplantation	Yes/No	Mild	No	7354 mg/dl	7.78	N/A	Kadosh^22^
19	M	48	African	N/A	Collapsing	*De novo*	DM 2, HTN, and CKD	Yes/Yes	Moderate	Yes	18 g/d	15.9	≥1 mo	Nlandu^71^
20	M	63	African	G1/G1	Collapsing	*De novo*	Acquired solitary kidney because of donation to his sister	Yes/Yes	Moderate	N/A	12.7 g/g	4.9	N/A	Wu^7^
21	F	64	African	G2/G2	Collapsing	*De novo*	Three patients had CKD stage 3A, four patients HTN, and three patients DM2	Yes/No	Moderate	N/A	4.6 g/g	4.2	N/A	
22	F	65	African	G1/G1	Collapsing	*De novo*		Yes/Yes	Critical	N/A	13.6 g/g	2.9	N/A	
23	M	44	African	G1/G1	Collapsing	*De novo*		Yes/Yes	Moderate	N/A	25 g/g	11.4	N/A	
24	M	37	African	G1/G2	Collapsing	*De novo*		Yes/Yes	Critical	N/A	3+ protein	9	N/A	
25	M	56	African	G1/G1	Collapsing	*De novo*		Yes/Yes	Moderate	N/A	3.6 g/g	6.7	N/A	
26	M	46	African	G1/G1	Collapsing	*De novo*	Obesity and OSA	Yes/Yes	Mild	Yes	10.376 g/d	19.9	≤1 mo	Peleg^72^
27	M	54	African	N/A	Collapsing	*De novo*	DM2, HTN, and smoking	Yes/No	Moderate	No	16 g/g	4.67	≤1 mo	Gupta^65^
28	M	54	African	N/A	Collapsing	*De novo*	HTN, obesity, and CKD G2	Yes/Yes	Mild	No	359 mg/mmol	13.59	≤1 mo	Noble^73^
29	F	28	African	G1/G1	Collapsing	*De novo*	Asthma	Yes/Yes	Severe	No	2 g/g	6.5	N/A	Magoon^11^
30	M	56	African	G1/G2	Collapsing	*De novo*	Uncontrolled HTN and CKD 3	Yes/Yes	Severe	No	>21 g/g	7.72	N/A	
31	M	57	African	N/A	Collapsing	*De novo*	Not known	Yes/Yes	Severe	No	14,865 mg/g	10.2	2 mo	Malik^74^
32	M	53	African	G1/G1	Collapsing	*De novo*	HTN and acute cardiac failure	Yes/No	Critical	No	1870 mg/mmol	2.19	≤1 mo	Couturier^75^
33	M	53	African	G1/G2	Collapsing	*De novo*	HTN and untreated chronic hepatitis B	Yes/No	Severe	No	2.65 g/d	5.98	≤1 mo	
34	M	65	African	G1/G2	Collapsing	*De novo*	HTN, DM2, DLP, and CKD 3A	Yes/Yes	Severe	Yes	15.8 g/g	6.5	N/A	Roy^76^
35	M	79	African	N/A	Collapsing	*De novo*	Hemorrhage stroke, MGUS, CKD 3 due to HTN	Yes/Yes	Mild	Yes	11.4 g/g	2.53	5 d	Gaillard^77^
36	F	50	African	G1/G1	Collapsing	*De novo*	HTN, hypothyroidism, depression, OSA, and obesity	Yes/Yes	Mild	No	12.5 g/g	6.08	≥1 mo	Meliambro^78^
37	F	38	African	N/A	Collapsing	*De novo*	Morbid obesity, status post gastric bypass, DM2, asthma, and a sister with membranous nephritis	Yes/No	Moderate	No	6.7 g/d	7.08	N/A	Tancredi^79^
38	F	61	African	N/A	Collapsing	*De novo*	HTN, obesity, and DM2	N/A	Severe	N/A	14 g/d	N/A	N/A	Gambella^80^
39	M	45	African	N/A	Collapsing	*De novo*	HTN and DM2	N/A	Moderate	N/A	30 g/d	N/A	N/A	
40	F	49	African	N/A	Collapsing	*De novo*	CKD 4, HTN, DM 2, heart failure, and obesity	Yes/Yes	Mild	No	19 g/g	7.17	≤1 mo	Kesiena^81^
41	F	21	African	G2/G0	Collapsing	*De novo*	SLE and active class IV lupus nephritis	Yes/No	Mild	Yes	6.0 g/g	1.3	≤1 mo	Masset^38^
42	M	52	African	G1/G1	Collapsing	*De novo*	N/A	Yes/Yes	N/A	N/A	1.7 g/g	2.68	10 mo	Nystrom^12^
43	M	44	African	G1/G1	Collapsing	*De novo*	N/A	Yes/N/A	N/A	N/A	14 g/g	1.9	N/A	
44	M	37	African	G1/G1	Collapsing	*De novo*	N/A	No/No	N/A	N/A	13 g/g	2.8	2 mo	
45	M	42	African	G2/G2	Collapsing	*De novo*	N/A	Yes/Yes	N/A	N/A	1.4 g/g	11.3	≤1 mo	
46	M	57	African	G1/G1	Collapsing	*De novo*	N/A	Yes/Yes	N/A	N/A	N/A	16.1	9 d	
47	M	60	African	G1/G2	Collapsing	*De novo*	N/A	Yes/No	N/A	N/A	7.9 g/g	6.1	≤1 mo	
48	M	58	African	G0/G0	Collapsing	*De novo*	N/A	Yes/Yes	N/A	N/A	6 g/g	3.53	≤1 mo	
49	M	49	African	N/A	Collapsing	*De novo*	Multifactorial liver cirrhosis (alcoholic and 1b genotype HCV infection) and active cocaine	Yes/No	Mild	Yes	22 g/12 h	2.27	≤1 mo	Papalia^82^
50	N/A	N/A	African	N/A	Collapsing	N/A	N/A	N/A	Critical	N/A	8.5 g/g	N/A	Postmortem	Santoriello^4^
51	M	46	African	G1/G1	Collapsing	*De novo*	HTN	Yes/Yes	Critical	No	13.7 g/g	8.7	2 wk	Akilesh^8^
52	F	60	African	N/A	Collapsing	*De novo*	HTN	Yes/N/A	Asymptomatic	No	21 g/g	5.7	4 wk	
53	F	58	African	N/A	Collapsing	*De novo*	HTN	Yes/Yes	Asymptomatic	No	20 g/g	10.2	8 d	
54	M	58	African	N/A	Collapsing^b^	*De novo*	Non-HTN and DM	Yes/Yes	Severe	No	4 g/d	11.3	4 d	
55	M	47	African	N/A	Collapsing^b^	*De novo*	HTN	Yes/Yes	Asymptomatic	No	7.6 g/g	6.6	25 d	
56	F	63	African	N/A	Collapsing^b^	*De novo*	HTN and adenocarcinoma	Yes/Yes	Mild	No	20 g/g	6	10–14 d	
57	M	56	African	N/A	Collapsing	*De novo*	Hyperlipidemia	Yes/Yes	Mild	No	15 g/d	4.97	N/A	Hale^83^
58	M	58	African	G1/G1	Collapsing	*De novo*	HTN	Yes/Yes	Moderate	N/A	9.3 g/d	21.5	N/A	Saleem^84^
59	F	29	African	G1/G1	Collapsing	Relapsing	Sickle cell disease, FSGS collapsing	Yes/No	Moderate	Yes	28 g/d	3.6	N/A	
60	M	53	African	G1/G1	Collapsing	*De novo*	HTN, new diagnosis of HIV, and syphilis	Yes/No	Mild	N/A	5.6 g/d	3.2	N/A	
61	N/A	42	African	N/A	Collapsing	*De novo*	Not known	Yes/Yes	Mild	N/A	5.6 g	12.5	N/A	Pendyala^85^
62	N/A	50	African	N/A	Collapsing	*De novo*	HTN	Yes/Yes	Mild	N/A	4.8 g	14	N/A	
63	N/A	55	African	N/A	Collapsing	*De novo*	Not known	Yes/Yes	Mild	N/A	6.2 g	9.6	N/A	
64	F	48	African	N/A	Collapsing	*De novo*	HTN and CKD	Yes/No	Mild	N/A	6.15 g/g	9.9	N/A	Akrawi^86^
65	F	32	African	High risk	Collapsing	*De novo*	LN class 3 + class V partial remission, SLE, and current pregnancy (12 wk)	Yes/No	Mild	Yes	13.6 g/d	4.7	N/A	Santosh^87^
66	F	57	African	N/A	Collapsing	*De novo*	DM2, HTN, and small cell lung cancer	Yes/No	Mild	Yes	12 g/g	1.96	N/A	Spinella^88^
67	F	51	African	N/A	Collapsing	*De novo*	Not known	Yes/No	Moderate	Yes	3.0 g/g	5.3	N/A	Zemke^89^
68	F	42	African	N/A	Collapsing	*De novo*	HTN and DM	Yes/Yes	Moderate	N/A	15.4 g/g	12.7	N/A	Scherchan^90^
69	M	48	African	N/A	Collapsing	*De novo*	Not known	Yes/No	N/A	No	1.4 g/g	4.8	N/A	Gallagher^91^
70	M	54	African	12 Patients at high risk	Collapsing	*De novo*	HTN and prostate carcinoma	Yes/Yes	Moderate	Yes	20 g/g or g/d	5.1	≤2 wk	Kudose^9^
71	M	66	African		Collapsing	*De novo*	HTN, obesity, and CKD	Yes/Yes	Mild	No	10 g/g	7.0	≤2 wk	
72	M	57	African		Collapsing	*De novo*	Not known	Yes/Yes	Moderate	Yes	10 g/g	3.0	1–2 mo	
73	M	68	African		Collapsing	*De novo*	HTN, DM, obesity, gout, BPH, and CKD	Yes/No	Moderate	Yes	6.6 g/g	6.8	≤2 wk	
74	M	64	African		Collapsing	*De novo*	HTN and CKD	No/Yes	N/A hospitalized	No	0.8 g/g	1.8	4 mo	
75	F	58	African		Collapsing	*De novo*	HTN	Yes/Yes	Moderate	Yes	4 g/g	7.4	≤2 wk	
76	M	52	African		Collapsing	*De novo*	HTN, DM, and carcinoid tumor	Yes/No	Moderate	Yes	30 g/g	10.6	≤2 wk	
77	M	55	African		Collapsing	*De novo*	HTN and obesity	Yes/No	Mild	Yes	18 g/g	2.6	≤2 wk	
78	F	56	African		Collapsing	*De novo*	HTN, DM, and obesity	Yes/No	Severe	No	3 g/g	3.4	≤2 wk	
79	M	57	African		Collapsing	*De novo*	HTN, obesity, and CKD	Yes/No	Mild	Yes	11 g/g	2.5	At 1 mo	
80	F	37	African		Collapsing	*De novo*	HTN, DM, and obesity	Yes/No	Mild	No	23 g/g	4.5	≤2 wk after resolution	
81	F	72	African		Collapsing	*De novo*	HTN, obesity, and CAD	Yes/Yes	Mild	No	13 g/g	11.5	≤2 wk	
82	F	59	African		Collapsing	*De novo*	HTN, obesity, untreated HCV, RA, and CKD	Yes/Yes	Severe	Yes	5 g/g	3.8	3 mo	
83	F	54	African		Collapsing	*De novo*	HTN, SLE, CVA, and CKD	Yes/Yes	Mild	Yes	2.2 g/g	12.9	≤2 wk	
84	M	52	African		Collapsing	*De novo*	HTN, DM, and ethanol abuse	Yes/Yes	Critical	No	3+ protein	31	≤2 wk	
85	M	35	African		Collapsing	*De novo*	Obesity and CNS vasculitis	Yes/Yes	Critical	Yes	16 g/g	10.9	3 wk	
86	F	50	Hispanic donor African recipient	Transplanted kidney G1/G0, the recipient was G1/G1	Collapsing	*De novo*	Two kidney transplants, the last was a deceased Hispanic donor. Banff 1B acute cellular rejection and CKD stage 4.	Yes/No	Moderate	No	6.11 g/d	5.99	≤2 wk	Shetty^10^
87	M	29	African	Low-risk G0/G2 genotype donor, the recipient was G0/G0	Collapsing	*De novo*	Urinary schistosomiasis, kidney transplant from a deceased donor—ABMR diagnosis	Yes/N/A	Mild	No	0.8 g/mmol	6.03	7 d	Lazareth^39^
88	M	16	African	N/A	Collapsing	*De novo*	CVA and CKD due to microscopic polyangiitis, living-related donor kidney transplant	Yes/No	Mild	Yes	Prot 17 mg/mg Album 8.8 mg/mg	4.7	13 d	Levenson^92^
89	M	49	African	G2/G2 donor recipient N/A	NOS	*De novo*	HTN associated with hypertrophic cardiomyopathy, kidney transplant	Yes/No	Moderate	Yes	3270 mg/g	2.17	≤1 mo	Oniszczuk^93^
90	M	45	African	N/A	Collapsing	*De novo*	DM2, obesity, CKD secondary to malignant hypertension, living donor transplant	Yes/Yes	Severe	Yes	8.0 g/g	10.27	N/A Autopsy	Noble^73^
91	M	36	African	G1/G2	NOS	*De novo*	HTN, DM, and obesity	Yes/No	Moderate	No	4.9 g/g	5.4	2–3 wk	Kudose^9^
92	F	52	African	N/A	NOS	*De novo*	Previously healthy	Yes/Yes	Critical	Yes	5.8 g/d	2.0	≥1 mo	Shabaka^94^
93	M	59	African	N/A	NOS	*De novo*	HTN and DM	Yes/N/A	Mild	No	>12 g/d	11.9	11 d	Akilesh^8^
94	F	43	African	N/A	Tip	*De novo*	Not known	Yes/No	Moderate	No	13.445 g/d	1.6	N/A	Afonso^95^

*APOL1*, Apolipoprotein L gen 1; COVID-19, coronavirus disease 2019; F, female; SLE, systemic lupus erythematosus; APS, antiphospholipid syndrome; M, male; Urine Protein-Creatinine Ratio (g/g) or 24-hours urine protein (g/d) or dipstick proteinuria; N/A, not available; HTN, hypertension; DM, diabetes mellitus; AFib, atrial fibrillation; DLP, dyslipidemia; OSA, obstructive sleep apnea; GERD, gastroesophageal reflux disease; MGUS, monoclonal gammopathy of unknown significance; HCV, hepatitis C virus; LN, lupus nephritis; BPH, benign prostatic hyperplasia; CAD, coronary artery disease; RA, rheumatoid arthritis; CVA, cerebrovascular accident; CNS, central nervous system; ABMR, antibody-mediated rejection; NOS, not otherwise specified.

a The term “African American” included Black people, including patients outside of the United States, on the basis of the descriptions in the references cited.

b TMA, thrombotic microangiopathy.

In 59 patients, hypertension was found to be the most frequent comorbidity. CKD was present in 25 patients, type 2 diabetes in 25, obesity in 21, and systemic lupus erythematosus in five (three with nonactive disease, one on partial remission, and one with active nephritis); four patients had untreated hepatitis (three with hepatitis C and one with hepatitis B); and two patients had HIV, one controlled and the other with active disease. The remaining nine patients has no comorbidities.

##### Renal Findings

In AA patients, FSGS was reported in native kidneys (n=89), one of those was from autopsy; the reminding cases of FSGS were from kidney allografts (n=5). Most of the biopsies were performed within 4 weeks of a positive SARS-CoV-2 PCR test (ranging from 5 days to 10 months). *De novo* FSGS was observed in 92 patients; relapsing collapsing FSGS was observed in one patient; and it was not specified in the remaining patient. Among the 94 AA patients with COVID-19 and FSGS, 89 had the collapsing variant, four had NOS, and the remaining one had tip (Figure 1B). In addition, three patients had thrombotic microangiopathy and collapsing glomerulopathy.

Of all 94 AA patients with FSGS and COVID-19, 88 patients had AKI, and no information was provided for three patients. The mean creatinine was 7.81±5.47 mg/dl, with a median of 6.6 mg/dl (range 1.30–31 mg/dl). Of the 94 patients, 50 required hemodialysis, and in seven patients, this was not specified.

Nephrotic range proteinuria was reported in 77 of the 94 patients. Of the remaining 17 patients, 15 had subnephrotic range proteinuria, one had +3 protein on a dipstick, and the remaining patient had no information available (autopsy patient).

##### Type of FSGS and *APOL1* Status

Of 94 AA patients, five had noncollapsing FSGS, and all the remaining 89 AA patients had collapsing FSGS (Figure 1B). *APOL1* status was reported in 55 of the 94 AA patients (Figure 2B).

In noncollapsing FSGS, four of five patients had the NOS variant and one had the tip variant. *APOL1* alleles were reported in two of five patients. In the NOS variant, two of four patients had *APOL1* genotyping: one with high-risk native kidney G1/G2 and the other with donor high-risk G2/G2; the recipient information, however, was not available; and the remaining two patients had no information available.

Of the 89 AA patients with collapsing FSGS, 53 were genotyped (Figure 2C). Forty-eight of them had a high-risk variant, all from native kidneys: 22 G1/G1, 10 G1/G2, 2 G2/G2, and one G1^GM^/G1^G+^. The remaining 13 patients had high-risk alleles that, however, were not specified. Five of 53 patients with collapsing FSGS had low-risk variants: G1/G0, G2/G0, and one without risk G0/G0. The remaining two patients had kidney transplants: one with donor low-risk G0/G2 and the other recipient G0/G0. The remaining patient had been transplanted with a combination of alleles: The African ancestry recipient had high-risk G1/G1, and the Hispanic donor had low-risk G1/G0 (Figure 2D, Table 2).

## Discussion

In this review of the literature on FSGS associated with COVID-19, we found only 19 patients reported as non–African American. Of note, 15 of the 19 patients had AKI, and seven required dialysis. In this regard, non-AA patients present with AKI similar to African American patients, although the reported creatinine values were not as high. Nephrotic range proteinuria was found in non-AA similar to AA patients. The relatively rare occurrence of FSGS in non–African American patients is in contrast with that in African American patients where this entity is found much more frequently in COVID-19–afflicted patients. We included data from 94 AA patients in this review, but this figure is clearly underestimated because we did not include, by our study design, studies that reported a series of patients with FSGS associated with COVID-19 without individual data that could be used for our analysis. For instance, Ferlicot *et al.*^2^ reported 17 patients with FSGS but did not specify the race. May *et al.*^1^ reported the largest multicenter retrospective cohort, including 107 genotyped patients. Giannini *et al.*^6^ reported 56 patients, but AA and Hispanic patients' characteristics were combined.

In addition to these reports, in an oral presentation during the last ASN conference (November 2022), Pourmehdi *et al.*^32^ reported the largest cohort in North America of kidney biopsies from fatal cases of COVID-19 (from April 2020 to July 2021). Unfortunately, of their 40 patients with COVID-19 with collapsing FSGS, none of them were genotyped for *APOL1*, and we were not able to include them because of the limited data provided in the abstract. We would like to highlight, however, that 40 of the 82 patients with COVID-19 had collapsing FSGS and that as many as 26 of these patients were non-AA. This large number of non-AA patients in this preliminary report is indeed surprising. This likely reflects, in part, the demographics of the overall Texas population with a predominance of Hispanic and White patients. Notwithstanding the large proportion of non-AA patients in this study is in contrast to our findings of only 19 patients worldwide, and of those patients, only 11 had collapsing FSGS. Other pathologic patterns of FSGS found were NOS (*n*=5), tip (*n*=2), and perihilar (*n*=1) (Table 1). This spectrum of patterns is in contrast with AA patients where the collapsing FSGS variant is, by far, the dominant pattern (89 of the 94 patients) (cf. Figure 1, A and B).

The proportion of African genetic ancestry among Hispanic/Latino patients has been associated with CKD risk.^33,34^ It is believed that because of the trans-Atlantic slave trade, there has been dispersion of high-risk *APOL1* genotype into Latin American and Caribbean populations.^20^ Self-identified race or ethnicity is not a reliable criterion to exclude the possibility that individuals carry *APOL1* risk alleles.^35^ Unfortunately, in research, unintentional racism might have a role in self-reported cases of race because of a taxonomy that categorized phenotypically defined humans.^36^ This is likely relevant to the *APOL1* status reported in non-AA patients with FSGS associated with COVID-19 in our review of the literature. We found that in non-AA patients, information on *APOL1* was found only for six patients (Figure 2A). Three of them were part of the 11 patients with collapsing FSGS, and all three had *APOL1* high-risk variants. Of the remaining eight patients, one had low-risk variants. It is well-known that the predominance of the collapsing variant in AA patients is attributable to the almost universal presence of high-risk variants for *APOL1* in AA patients. Consistent with this notion, 48 of the 53 AA patients with collapsing FSGS in whom *APOL1* was performed had high-risk variants (Figure 2B). Of note, however, there were five AA patients with collapsing FSGS associated with low-risk variants. In non-AA patients, we found one of four patients with the collapsing variant who also had low-risk variants (Figure 2A). The patients with collapsing FSGS with low-risk variants might provide clues for pathophysiological mechanisms involved in the development of FSGS in patients with COVID-19. Collapsing FSGS may occur even in lower-risk heterozygous *APOL1* variants.^37,38^

In patients with a low-risk genotype, but with at least one G2 risk allele, combined with viral infection and a hyperinflammatory state, podocyte damage may increase.^39^ Among individuals with collapsing glomerulopathy and *APOL1* risk alleles, other gene–gene interactions may contribute to glomerulopathy.^40^ Systemic inflammation due to cytokine storm and interferon-mediated inflammatory signaling has been incriminated in COVID-19–associated FSGS.^12^ This is similar to other high interferon states that have been associated with collapsing FSGS, such as HIV, SLE, and treatment with interferon of hepatitis B and other conditions.^12,41–44^ Renal tropism and direct viral infection^45–47^ were speculated as the triggers for collapsing FSGS.^22,48^ This speculation of viral renal tropism is difficult to support given the fact that SARS-CoV-2 kidney invasion in patients with COVID-19 is usually not demonstrable.^49,50^ Other possible pathological mechanisms like “the three hit hypothesis for collapsing glomerulopathy” in the context of viral infection where the first hit is the high-risk *APOL1* genotype, second hit being the inflammatory reaction and cytokines and third hit, modifier genes.^46^ COVID-19–associated nephropathy without the high-risk *APOL1* genotype suggests other mechanisms triggered by viral infections that might be independent of *APOL1* genotype.^12,37–39,51^ A role for non–renal cell *APOL1* variant expression in COVID-19–associated FSGS has also been suggested.^10,52^ Regarding the similarities between COVAN and HIV-associated nephropathy (HIVAN), some studies found a high percentage (72%) of *APOL1* high-risk alleles in HIVAN-associated FSGS.^53^ The effect of carrying two *APOL1* risk alleles explains 18% of FSGS in general and 35% of HIVAN.^28^

Regardless of the mechanisms, the findings that there are cases of collapsing FSGS in AA and non-AA patients with COVID-19 and low risk *APOL1* variants suggest that it would be misleading to assume that everyone with this disease has *APOL1* high-risk variants. We corroborate that in the AA patients with collapsing FSGS, the percentage of patients with COVID-19 and high-risk alleles is more than 90%.^1,2,9^ On comparison, among the 11 non-AA patients with collapsing FSGS with known *APOL1* status, we found three of four (75%) with high-risk variants (Figure 2). Considering the abstract by Pourmehdi *et al.*^32^ reporting 26 of 40 non-AA patients with collapsing FSGS and COVID-19, it seems likely that this lesion occurs much more frequently in patients without high-risk *APOL1* variants than in AA patients. There are four reported cases of collapsing FSGS in Hispanic patients: one from Mexico with *APOL1* high-risk G2/G2, during infection with COVID-19, had a relapse of the disease^8^ and three patients had *de novo* glomerulopathy—one with *APOL1* high-risk (G1/G1), one low-risk G0/G0,^12^ and the other transplant patient not genotyped.^54^ *APOL1* testing is available in some clinical laboratories and should be increasingly used in the evaluation of kidney disease and most definitely, in our opinion, in patients with FSGS associated with viral infections, regardless of the reported race. A significant bias has been noted in the field of genome-wide association studies, with the majority of discovery efforts conducted in populations of European ancestry. By contrast, individuals of African or Latin American ancestry accounted for only 4.2% of samples analyzed.^55^

Follow-up of renal function in COVID-19 patients with FSGS is limited, in general, and similarly, very little information was available for the 19 non-AA patients reviewed. Some reports indicated that half of the patients with FSGS who initially required dialysis achieved dialysis independence^6,9^ while others required dialysis at follow-up, but not at presentation.^6^ Some patients with collapsing FSGS and COVID-19 developed ESKD and/or died, which is similar to collapsing FSGS without COVID-19.^6,56^ In a recent review by Giannini *et al.*,^6^ nearly all patients with COVAN had advanced CKD at follow-up, with most showing no remission or disease progression. Similarly, in HIVAN, ESKD developed in more than half of the patients.^57,58^

In summary, non-African American patients, collapsing FSGS has been reported rarely as a complication of COVID-19, and it can be associated with high-risk and a minority of low-risk APOL1 variants. In African American patients, more than 90% of cases reported are associated with high-risk *APOL1* variants but it can also occur with low-risk variants. *APOL1* genotyping in non-AA patients associated with COVID-19 was rarely performed, and ancestry was not always specified in the reviewed literature. Considering potential issues with race admixture and inaccuracy of self-reported race and also to avoid racial bias, it seems appropriate that *APOL1* testing, which is increasingly available, be considered in all patients with FSGS associated with COVID-19.
